# Urokinase Attenuates Pulmonary Thromboembolism in an Animal Model by Inhibition of Inflammatory Response

**DOI:** 10.1155/2018/6941368

**Published:** 2018-12-25

**Authors:** Ying Shi, Zhirong Zhang, Danli Cai, Jing Kuang, Shuifang Jin, Chen Zhu, Yingying Shen, Wen Feng, Songmin Ying, Lingcong Wang

**Affiliations:** ^1^Department ICU of the First Affiliated Hospital, Zhejiang Chinese Medical University, Hangzhou, China; ^2^Zhejiang Chinese Medical University, Hangzhou, China; ^3^Department of Pharmacology, Zhejiang University School of Medicine, Hangzhou, China

## Abstract

Inflammatory response is an important determining factor for the mortality of patients with pulmonary thromboembolism. Inflammatory mediators can promote thrombus formation and increase hemodynamic instability. Urokinase is a commonly used drug for the treatment of PTE. The effect of urokinase on inflammatory reaction in PTE is still unclear. Our study was aimed at evaluating the effects of the intervention of urokinase and urokinase combined with aspirin in PTE rats. Results revealed that a large amount of infiltrated inflammatory cells surrounding the bronchus, vessels, and pulmonary mesenchyme, and even pulmonary abscess were observed in the PTE rats. CX3CL1/CX3CR1 coexpression, CX3CL1/NF-*κ*B coexpression, and TXA2 were significantly higher. After treatment with urokinase, pulmonary embolism was partially dissolved and inflammatory cell infiltration was significantly reduced. The expression of TNNI3, BNP, D2D, PASP, PADP, PAMP, and TXA2, as well as CX3CL1/CX3CR1 coexpression and CX3CL1/NF-*κ*B coexpression were significantly lowered. Aspirin showed no synergistic action. Therefore, these findings suggested the occurrence of inflammation during the process of PTE in rats. Urokinase treatment reduced the inflammatory response.

## 1. Introduction

Acute pulmonary embolism (PTE) leads to a rapid hemodynamic collapse and death. It is potentially a life-threatening disease with significant morbidity and fatal outcomes [1]. As a result of pulmonary hypertension, hemodynamically unstable patients are at high risk of death from worsening RV failure and cardiogenic shock, with a hospital mortality rate of >15% [2]. Approximately one quarter of the hemodynamically stable patients with PTE show the imaging or biomarker evidence of RV dilatation or dysfunction, with mortality rates ranging from 3% to 15% [3]. Hence, new interventions are urgently needed.

Inflammatory response is an important determining factor for the mortality of patients with pulmonary embolism. Leukocytosis and SIRS are important factors for determining the short-term outcomes in PTE patients clinically [4, 5]. Ischemia and pulmonary hypertension associated with PTE are sufficient to induce the expression of proinflammatory mediators, such as chemokines, and establish a proinflammatory environment in the ischemic lung even before reperfusion [6]. Chemokines massively recruit monocytes, NT cells, and T cells. Inflammatory reaction promotes thrombosis and pulmonary hypertension, and it is associated with poor prognosis [7, 8]. Persistent inflammatory response is also involved in pulmonary vascular remodeling, leading to chronic thromboembolic pulmonary hypertension (CTEPH) [9, 10].

Our previous studies have reported that CX3CL1, CX3CR1, and NF-*κ*B are significantly increased after pulmonary embolism, and NF-*κ*B signaling pathway and CX3CL1/CX3CR1 are perhaps directly involved in the whole inflammatory response of pulmonary embolism [11–14]. Moreover, aspirin can significantly reduce CX3CL1, CX3CR1, and NF-*κ*B in rats with pulmonary thromboembolism, and subsequently control the inflammatory response. Urokinase is the most commonly used drug for the treatment of PTE. The effect of urokinase on inflammatory reaction in PTE is still unclear. Hence, the synergistic action of aspirin remains obscure. We also found that lipopolysaccharide- (LPS-) extracellular signal-regulated protein kinase (ERK), nuclear factor-*κ*B (NF-*κ*B), and CX3CL1 signal pathways exist in human bronchial epithelial cells [14]. So we choose urokinase to interfere with the CX3CL1, CX3CR1, and NF-*κ*B pathways.

In this study, we specifically investigated the anti-inflammatory effects of urokinase and urokinase combined with aspirin. PTE rats were treated with urokinase and urokinase combined with aspirin, respectively, in this study. Pulmonary tissues were examined by H&E staining. CX3CL1/CX3CR1 coexpression, CX3CL1/NF-*κ*B coexpression, and TXA2 were used to observe the inflammatory response. Pulmonary systolic pressure (PASP), diastolic pressure (PADP), and mean pressure (PAMP) were monitored to observe the effects of inflammatory response on pulmonary arterial pressure. TNNI3, BNP, and D2D were used to determine the severity and prognosis of PTE.

## 2. Materials and Methods

### 2.1. Animals

The study included male Sprague-Dawley (SD) rats weighing about 200 g. The rats were obtained from Shanghai Laboratory Animal Center (SLAC) Laboratory Animal Co. Ltd. and Beijing Wei Tong Li Hua Laboratory Animal Co. Ltd. Specific-pathogen-free (SPF) rats were used in this study. The Medical Experimental Animal Management Committee of Zhejiang, China, as well as the ethics committee of Zhejiang Chinese Medical University (Hangzhou, China) approved this study, and the ARRIVE Guidelines were followed for animal studies.

### 2.2. Grouping and Model Preparation

A total of 50 male SPF SD rats were randomly assigned into five groups according to the computer generated random number table (*n* = 10 rats per group): control group (Group control), mock control group (Group mock control), pulmonary thromboembolism group (Group PTE), pulmonary thromboembolism combined with urokinase group (Group PTE + UK), and pulmonary thromboembolism combined with urokinase and aspirin group (Group PTE + UK + aspirin).

#### 2.2.1. Pulmonary Thromboembolism Model Preparation

One day before operation, 0.2 ml of venous blood was withdrawn from the caudal vein and incubated in a 37°C water bath overnight. The concretionary thrombus was taken out with a syringe to prepare 30 emboli of 2 mm ^∗^ 1 mm size, and these were further placed into a 2 ml syringe. The rats were anesthetized using ether. The right jugular vein was separated, and the puncture needle was placed. The prepared embolus was pushed into the general jugular vein through the puncture needle, followed by pushing 1 ml of saline to prevent the embolus from staying in the tube or jugular vein. Finally, the wound was sutured after the bleeding was stopped. In the mock control group, after administration of abdominal anesthesia, the right jugular vein was separated and then 1 ml of saline was quickly injected into the vein.

The rats were intragastrically administered with drugs on the day before the surgery and 40 minutes before the model establishment. The rats in the PTE + UK + aspirin group were administered with 300 mg/kg aspirin intragastrically (Nanjing BAIJINGYU Pharmaceutical Co. Ltd., Nanjing, China) as described in the previous study [11]. Except for the control group, the rats in the other groups were intragastrically administered with equal amounts of normal saline every day. After the establishment of the pulmonary thromboembolism model, the rats in the PTE + UK and PTE + UK + aspirin groups received intravenous injections of 20,000 IU/kg urokinase (ND Pharmaceuticals Co. Ltd., Nanjing, China) in 2 ml of normal saline within 0.5 hours. The rats in the control group did not accept any intervention.

### 2.3. Pulmonary Arterial Pressure Measurement

After six hours of model establishment, the animals were anesthetized again, and pulmonary artery pressures were measured. The PTE50 catheter was inserted into the pulmonary artery through the right ventricle. The other end of the catheter was connected to the pressure transducer. The functional experimental system (Chengdu Thai Union BL-420S-TyPTE) was used to record the stable pulmonary arterial pressure waveform for 3 minutes. The pulmonary artery systolic pressure (PASP), diastolic blood pressure (PADP), and mean pulmonary artery pressure (PAMP) were calculated in the offline status.

### 2.4. Detection of H&E and Determination of TXA2, BNP, TNNI3, and D2D

After the animals were anesthetized using ether, we euthanized the animals by taking off the animals' neck, and then the pulmonary tissues were obtained and blood tests were conducted. Thromboxane A2 (TXA2, Youersheng Science and Technology Co. Ltd., Wuhan, China), brain natriuretic peptide (BNP, Youersheng Science and Technology Co. Ltd., Wuhan, China), troponin I gene (TNNI3, Youersheng Science and Technology Co. Ltd., Wuhan, China), and D-Dimer (D2D, Youersheng Science and Technology Co. Ltd., Wuhan, China) levels in the rat serum were detected by a double-antibody sandwich enzyme-linked immunosorbent assay (ELISA). The specific experimental procedures used are based on our previously reported studies [11–13]. Pulmonary pathology examined was as follows: after the examination of the gross pathology of the lung, lung tissue was fixed by 10% formalin for 24 h. The paraffin-embedded sections were prepared and then stained with a hematoxylin-eosin (H&E) reagent.

### 2.5. CX3CL1/CX3CR1 Coexpression and CX3CL1/NF-*κ*B Coexpression of Pulmonary Tissues Were Detected by Laser Confocal Scanning

The thickness of 4 *μ*m was prepared by slicing after deparaffinization by xylene and rehydration by graded alcohol, and the antigen was then retrieved. The slides were incubated with primary antibodies (CX3CL1, Santa Cruz Biotechnology, Inc., sc-7227; NF-*κ*B/p65, Abcam, ab7970; CX3CR1, Abcam, ab8021) at 4°C overnight. After cooling for 30 minutes at room temperature and washing for 3 × 5 min with phosphate-buffered saline (PBS), the slides were incubated with a fluorescent secondary antibody mixture (Life Technologies, A21206) for 60 minutes at room temperature. The slices were washed again in PBS for 3 × 5 min. The cell nucleus was stained with DAPI (Sigma-Aldrich). Then, the slides were incubated for 10 minutes at room temperature. The slides were covered using glycerin in PBS and were then observed under an inverted microscope.

### 2.6. Statistical Analysis

Statistical analyses were performed using IBM SPSS version 21.0 statistical software for Windows. Data were expressed as means ± standard deviations. Multifactor analysis of variance (ANOVA) was used for comparison among the groups, and post hoc test of Fisher's Least Significant Difference (LSD) was used for comparison among the groups. *P* < 0.05 was considered to be statistically significant.

## 3. Results

### 3.1. The Inflammatory Response Existed in the PTE Process and Was Associated with NF-*κ*B Pathway and CX3CL1/CX3CR1

**Figure 1:**
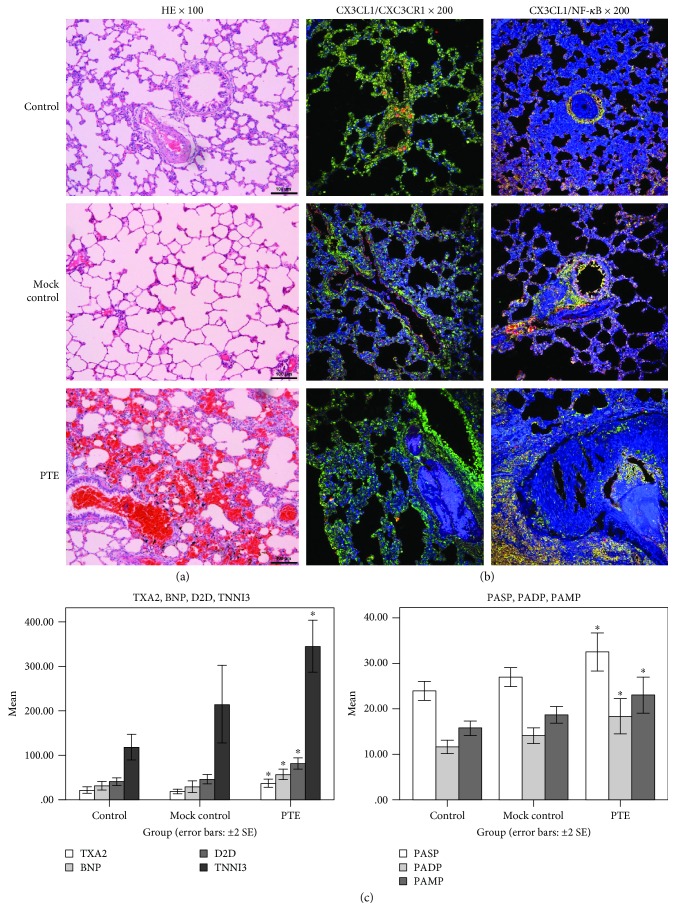


Examination of pulmonary tissues by H&E staining in PTE rats showed mixed and coagulated thrombi in the pulmonary artery. A large amount of infiltrated inflammatory cells surrounding the bronchus, vessels, and pulmonary mesenchyme, and even pulmonary abscess were observed. However, the structure of the pulmonary tissue appeared clear, and the alveolar structure appeared normal in the control rats. No obvious infiltration of inflammatory cells was observed in the pulmonary mesenchyme. Occasionally, inflammation was observed around the airways and vessels. No formation of thromboembolism was observed in the vessels. The results of mock control rats were similar to that of control rats (Figure 1(a)). CX3CL1/CX3CR1 coexpression, CX3CL1/NF-*κ*B coexpression (Figure 1(b)), TXA2, TNNI3, BNP, D2D, PASP, PADP, and PAMP (Figure 1(c), *P* < 0.05) in PTE rats were significantly higher. The experiment showed an inflammatory reaction during the PTE process and was related to the NF-*κ*B signaling pathway and CX3CL1/CX3CR1.

### 3.2. Urokinase Reduced Inflammation in PTE Rats

**Figure 2:**
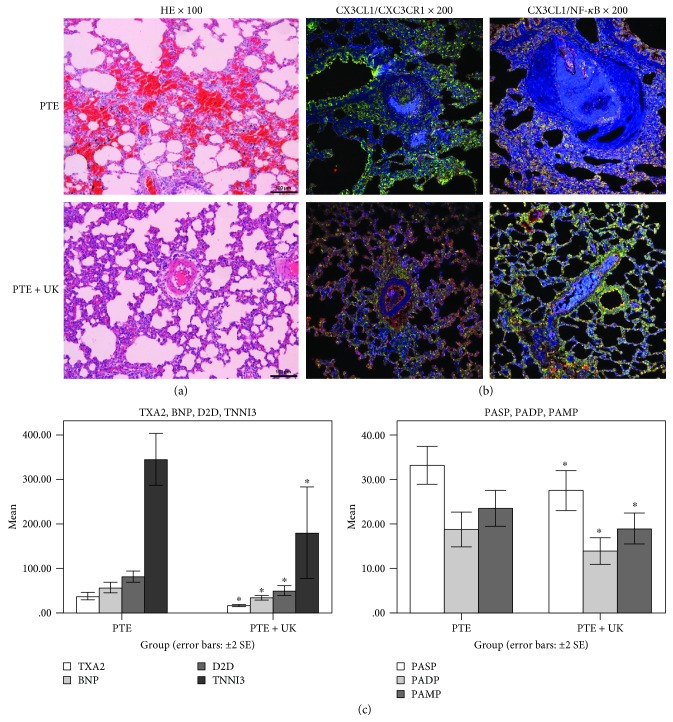


After urokinase treatment in PTE rats, pulmonary embolism was partially dissolved, and some vessels achieved recanalization in pulmonary tissues by H&E staining. No obvious thromboembolism was observed in the slices. Mild proliferation of vascular endothelial cells was observed, and the inflammatory reaction was similarly mild (Figure 2(a)). Coexpression of CX3CL1/NF-*κ*B and CX3CL1/CX3CR1 (Figure 2(b)) was obviously improved. TXA2, TNNI3, BNP, D2D, PASP, PADP, and PAMP (Figure 2(c), *P* < 0.05) were significantly lower. These results suggested that urokinase could improve the prognosis and modulate the inflammatory reaction.

### 3.3. Urokinase Combined with Aspirin Could Reduce the Inflammatory Response in PTE Rats

**Figure 3:**
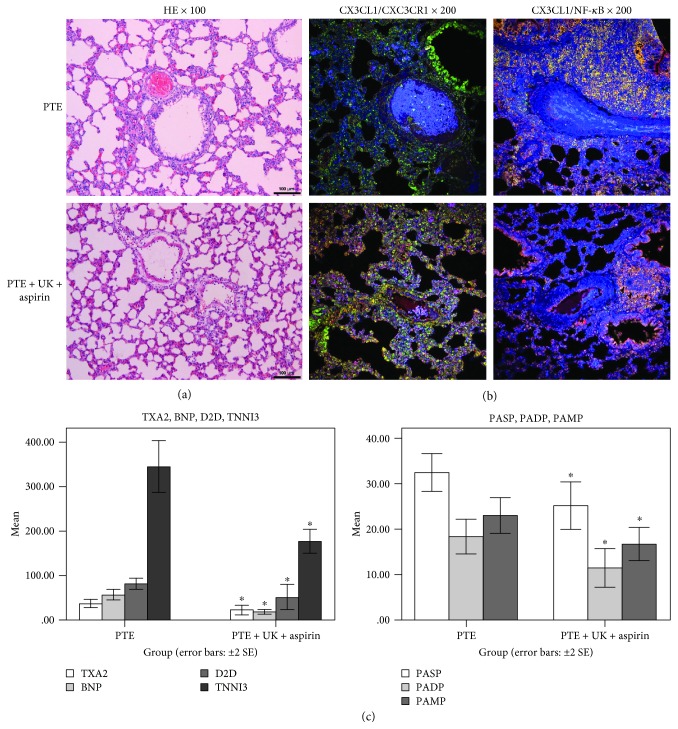


In view of the anti-inflammatory effect of aspirin [11–14], we chose urokinase combined with aspirin that might interfere with PTE. After treatment, pulmonary embolism was partly dissolved, and H&E staining revealed recanalization of some vessels in the pulmonary tissues. Mild proliferation of vascular endothelial cells was observed, and the inflammatory reaction remained similarly mild. H&E staining was similar to urokinase (Figure 3(a)). Coexpression of CX3CL1/NF-*κ*B (Figure 3(b)) and TXA2, TNNI3, BNP, D2D, PASP, PADP, and PAMP (Figure 3(c), *P* < 0.05) were significantly lower. Coexpression of CX3CL1/CX3CR1 was higher. Urokinase combined with aspirin could reduce the inflammation.

### 3.4. Aspirin Did Not Enhance the Anti-Inflammatory Function

**Figure 4:**
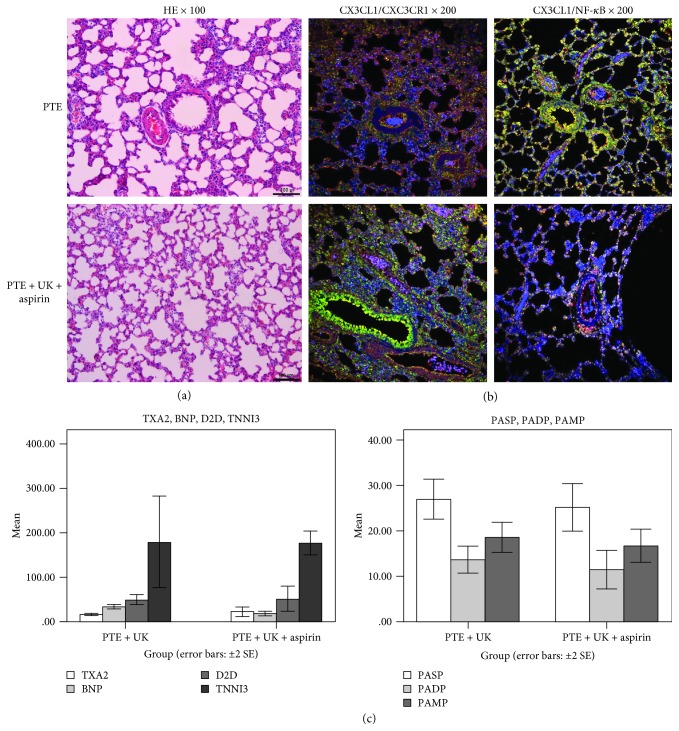


There were no significant differences in H&E staining, CX3CL1/NF-*κ*B coexpression, TXA2, TNNI3, BNP, D2D, PASP, PADP, and PAMP between the urokinase group and urokinase combined with aspirin group. Urokinase was superior in CX3CL1/CX3CR1 coexpression, and aspirin showed no synergistic action (Figure 4).

## 4. Discussion

Our study showed that inflammatory reaction was involved during the process of PTE, and it was associated with the NF-*κ*B signaling pathway and CX3CL1/CX3CR1. Urokinase could improve the inflammatory reaction in PTE. Aspirin did not enhance the anti-inflammatory function. Urokinase alone was superior to urokinase combined with aspirin in CX3CL1/CX3CR1 coexpression.

According to the results of previous study experiments [11–14], high-dose aspirin showed anti-inflammatory effects. Hence, our study has set up the urokinase combined with aspirin group. In this experiment, aspirin showed no enhancement of the anti-inflammatory function and the coexpression of CX3CL1/CX3CR1 has worsened. Considering the side effects of the drug, aspirin might not be used in combination. As the inflammatory reaction remains complex in PTE, this experiment studied only from a single signaling pathway. So, the above conclusions should be carefully considered.

More and more attention should be paid to study PTE inflammation [15, 16]. For example, statin therapy has the ability to decrease the incidence and recurrence of VTE and has the potential to decrease postthrombotic syndrome (PTS) through the anti-inflammatory effect [17, 18]. Few studies have discussed the anti-inflammatory effects of anticoagulant thrombolytic drugs. In the rabbit model with acute pulmonary embolism, urokinase decreased MCP-1 levels in the lung tissue or serum [19]. These findings were inconsistent with our study. In contrast, the expression of NF-*κ*B P65 protein in the cytoplasm and nucleus of alveolar macrophages, vascular endothelial cells, and alveolar and bronchial epithelial cells of PTE rabbits in the urokinase group was slightly higher than that in the PTE group. The acute inflammatory injury of the lung tissues after urokinase treatment showed no significant reduction [20]. The differences between the two studies include the usage of different animals, the size of emboli, and the dose of urokinase. We used 2 times more urokinase than the above study. Hence, we need to further study the anti-inflammatory effects with different dose concentrations of urokinase.

However, our study has some limitations. Firstly, although inflammatory reactions contribute to the aggravation of thrombus formation, the relationship between the two factors needs to be further explored. Secondly, only five groups of rats have been established in this study, but aspirin as a control group was not established as in our previous study [11–13]. This was done in order to decrease the unnecessary animal sacrifices.

Although an inflammatory reaction might not be the most important mechanism of PTE, it promotes the aggravation of embolism. Hence, it is necessary to further explore the relationship between the two. Urokinase can inhibit an inflammatory response, but whether the anti-inflammatory effect is dependent on the dose and whether other drugs need to be combined or not needs further research and discussion. In the future, effective drugs may be screened for clinical application. The drugs are not only thrombolytic, but they can also improve the inflammatory response and reduce the mortality.

## Data Availability

The data used to support the findings of this study are included within the article.

## Abbreviations

PTE: Acute pulmonary embolism
CTEPH: Chronic thromboembolic pulmonary hypertension
LPS: Lipopolysaccharide
ERK: Extracellular signal-regulated protein kinase
NF-*κ*B: Nuclear factor-*κ*B
PASP: Pulmonary systolic pressure
PADP: Diastolic pressure
PAMP: Mean pressure
TNNI: Troponin I gene
BNP: Brain natriuretic peptide
D2D: D-Dimer
SD: Sprague-Dawley
SPF: Specific-pathogen-free
TXA2: Thromboxane A2
ELISA: Enzyme-linked immunosorbent assay
H&E: Hematoxylin-eosin
PBS: Phosphate-buffered saline
LSD: Least significant difference
PTS: Postthrombotic syndrome
MCP-1: Monocyte chemotactic protein 1.

## References

[1] Konstantinides S. V., Torbicki A., Agnelli G. (2014). 2014 ESC guidelines on the diagnosis and management of acute pulmonary embolism. *European heart journal*.

[2] Marshall P. S., Tapson V., Jimenez D. (2015). Controversies in the management of life-threatening pulmonary embolism. *Seminars in Respiratory and Critical Care Medicine*.

[3] Kasper W., Konstantinides S., Geibel A. (1997). Management strategies and determinants of outcome in acute major pulmonary embolism: results of a multicenter registry. *Journal of the American College of Cardiology*.

[4] Kong T., Park Y. S., Lee H. S. (2017). Value of the delta neutrophil index for predicting 28-day mortality in patients with acute pulmonary embolism in the emergency department. *Shock*.

[5] Venetz C., Labarère J., Jiménez D., Aujesky D. (2013). White blood cell count and mortality in patients with acute pulmonary embolism. *American Journal of Hematology*.

[6] Zagorski J., Debelak J., Gellar M., Watts J. A., Kline J. A. (2003). Chemokines accumulate in the lungs of rats with severe pulmonary embolism induced by polystyrene microspheres. *The Journal of Immunology*.

[7] Saghazadeh A., Hafizi S., Rezaei N. (2015). Inflammation in venous thromboembolism: cause or consequence?. *International Immunopharmacology*.

[8] Saghazadeh A., Rezaei N. (2016). Inflammation as a cause of venous thromboembolism. *Critical Reviews in Oncology Hematology*.

[9] Lang I. M., Pesavento R., Bonderman D., Yuan J. X. (2013). Risk factors and basic mechanisms of chronic thromboembolic pulmonary hypertension: a current understanding. *European Respiratory Journal*.

[10] Quarck R., Wynants M., Verbeken E., Meyns B., Delcroix M. (2015). Contribution of inflammation and impaired angiogenesis to the pathobiology of chronic thromboembolic pulmonary hypertension. *European Respiratory Journal*.

[11] Wang L., Wu J., Zhang W. (2013). Effects of aspirin on the ERK and PI3K/Akt signaling pathways in rats with acute pulmonary embolism. *Molecular Medicine Reports*.

[12] Wang L. C., Jiang R. L., Zhang W., Wei L. L., Yang R. H. (2014). Effects of aspirin on the expression of nuclear factor-*κ*B in a rat model of acute pulmonary embolism. *World Journal of Emergency Medicine*.

[13] Wang L. C., Wu J. N., Xia G. L. (2014). Effect of aspirin on Fractalkine in rats with pulmonary embolism. *Tropical Journal of Pharmaceutical Research*.

[14] Jiang R. L., Wei L. L., Zhu M. F., Wu J. N., Wang L. C. (2016). Aspirin inhibits LPS-induced expression of PI3K/Akt, ERK, NF-*κ*B, CX3CL1, and MMPs in human bronchial epithelial cells. *Inflammation*.

[15] Ripplinger C. M., Kessinger C. W., Li C. (2012). Inflammation modulates murine venous thrombosis resolution in vivo: assessment by multimodal fluorescence molecular imaging. *Arteriosclerosis Thrombosis and Vascular Biology*.

[16] Wagner E. M., Sánchez J., McClintock J. Y., Jenkins J., Moldobaeva A. (2008). Inflammation and ischemia-induced lung angiogenesis. *American Journal of Physiology-lung Cellular and Molecular Physiology*.

[17] Khemasuwan D., Chae Y. K., Gupta S. (2011). Dose-related effect of statins in venous thrombosis risk reduction. *The American Journal of Medicine*.

[18] Rodriguez A. L., Wojcik B. M., Wrobleski S. K., Myers D. D., Wakefield T. W., Diaz J. A. (2012). Statins, inflammation and deep vein thrombosis: a systematic review. *Journal of Thrombosis and Thrombolysis*.

[19] Wu J. P., Sun X., Wu Q. (2013). Effect of low-molecular-weight heparin and urokinase on pulmonary arteries involved in pulmonary embolism. *Chinese Medical Journal*.

[20] Zhang D. X., Zhang L., Bai M. (2005). The expression levels of endothelin-1 and nuclear factor-kappa B in the lung tissue of acute pulmonary embolism and the effects of thrombolysis and dexamethasone. *Zhonghua Jie He He Hu Xi Za Zhi*.

